# Association of admission testosterone level with ST-segment resolution in male patients with ST-segment elevation myocardial infarction undergoing primary percutaneous coronary intervention

**DOI:** 10.1186/s12610-017-0058-7

**Published:** 2017-07-21

**Authors:** Ahmad Separham, Samad Ghaffari, Bahram Sohrabi, Naser Aslanabadi, Mozhgan Hadavi Bavil, Hasanali Lotfollahi

**Affiliations:** Cardiology Department, Cardiovascular Research Center, Tabriz University of Medical science, Madani Heart Center, Daneshgah Ave, Tabriz, Iran

**Keywords:** ST-segment elevation myocardial infarction, Serum testosterone, ST-segment resolution, Primary percutaneous coronary intervention, Infarctus du myocarde avec sus-décalage du segment ST, Testostérone sérique, Résolution du segment ST, Intervention coronarienne percutanée précoce

## Abstract

**Background:**

Low level of testosterone may be associated with cardiovascular diseases in men, as some evidence suggests a protective role for testosterone in cardiovascular system. Little is known about the possible role of serum testosterone in response to reperfusion therapy in ST-elevation myocardial infarction (STEMI) and its relationship with ST-segment recovery. The present study was conducted to evaluate the association of serum testosterone levels with ST-segment resolution following primary percutaneous coronary intervention (PPCI) in male patients with acute STEMI.

**Methods:**

Forty-eight men (mean age 54.55 ± 12.20) with STEMI undergoing PPCI were enrolled prospectively. Single-lead ST segment resolution in the lead with maximum baseline ST-elevation was measured and patients were divided into two groups according to the degree of ST-segment resolution: complete (> or =50%) or incomplete (<50%). The basic and demographic data of all patients, their left ventricular ejection fraction (LVEF) and laboratory findings including serum levels of free testosterone and cardiac enzymes were recorded along with angiographic finding and baseline TIMI (Thrombolysis in Myocardial Infarction) flow and also in-hospital complications and then these variables were compared between two groups.

**Results:**

A complete ST-resolution (≥50%) was observed in 72.9% of the patients. The serum levels of free testosterone (*P* = 0.04), peak cardiac troponin (*P* = 0.03) were significantly higher and hs-CRP (*P* = 0.02) were lower in patients with complete ST-resolution compared to those with incomplete ST-resolution. In-hospital complications were observed in 31.2% of patients. The patients with a lower baseline TIMI flow (*P* = 0.03) and those who developed complications (*P* = 0.04) had lower levels of free testosterone. A significant positive correlation was observed between the left ventricular function and serum levels of free testosterone (*P* = 0.01 and r = +0.362).

**Conclusion:**

This study suggests that in men with STEMI undergoing PPCI, higher serum levels of testosterone are associated with a better reperfusion response, fewer complications and a better left ventricular function.

**Electronic supplementary material:**

The online version of this article (doi:10.1186/s12610-017-0058-7) contains supplementary material, which is available to authorized users.

## Résumé

**Contexte:**

On considère qu’un faible taux de testostérone pourrait être associé aux maladies cardiovasculaires chez l’homme, car certains faits suggèrent un rôle protecteur de la testostérone sur la fonction cardiaque. On a très peu de connaissances sur le rôle possible des taux de testostérone sérique dans la réponse au traitement de reperfusion de l’infarctus du myocarde avec sus-décalage du segment ST, et de son implication dans la récupération du segment ST. Le but de cette étude était d’évaluer l’association entre les taux de testostérone et la résolution du segment ST qui suit une intervention coronarienne percutanée précoce (ICPP) chez les patients qui présentent un infarctus aigu du myocarde avec sus-décalage du segment ST.

**Matériel et Méthodes:**

Ont été enrôlés de façon prospective 48 hommes de 54.5 ± 12.2 ans d’âge moyen chez lesquels une ICPP était réalisée en raison d’un infarctus du myocarde avec sus-décalage du segment ST. La résolution du segment ST a été mesurée sur une seule dérivation choisie comme celle présentant l’élévation de base maximale du segment ST; les patients ont été répartis en 2 groupes selon le degré de résolution complète (≥ 50%) ou incomplète (<50%) du segment ST. Les données démographiques et les valeurs de base de chaque patient, leur fraction d’éjection ventriculaire gauche ainsi que les résultats des tests de laboratoire -incluant les taux sériques de testostérone libre et d’enzymes cardiaques- ont été enregistrés parallèlement aux données angiographiques et au flux de thrombolyse durant l’infarctus du myocarde (TIMI). Les complications survenues durant leur séjour hospitalier ont aussi été relevées. Ces différentes variables ont ensuite été comparées entre les 2 groupes de patients.

**Résultats:**

Une résolution complète du segment ST (≥ 50%) a été observée chez 72,9% des patients. Le taux sérique de testostérone libre (*P* = 0,04) et le pic de troponine cardiaque (*P* = 0,03) étaient significativement plus élevés, et la protéine C réactive (hs-CRP; *P* = 0,02) significativement plus basse chez les patients qui avaient une résolution complète du segment ST que chez ceux qui présentaient une résolution incomplète du segment ST. Des complications sont survenues chez 31,2% des patients au cours de leur séjour hospitalier. Les patients qui avaient un flux TIMI plus réduit (*P* = 0,03) et ceux qui avaient développé des complications (*P* = 0,04) présentaient des taux de testostérone libre plus bas. Une corrélation positive significative a été retrouvée entre la fonction ventriculaire gauche et les taux sériques de testostérone libre (*P* = 0.01 and r = +0.362).

**Conclusion:**

La présente étude suggère que, chez les hommes soumis à une intervention coronarienne percutanée précoce (ICPP) pour infarctus du myocarde avec sus-décalage du segment ST (STEMI), des taux sériques plus élevés de testostérone sont associés à une meilleure réponse à la reperfusion, à un moindre taux de complications et à une meilleure fonction du ventricule gauche.

## Background

Cardiovascular disease (CVD) is a major cause of mortality in older men. The increased risk of cardiovascular events in aging male may be related to low serum testosterone levels and hypogonadism [1–3]. Testosterone is a strong vasodilator of coronary arteries with the calcium channel antagonist effect [4]. Testosterone has positive effects on the angina threshold, especially in men with low basal testosterone levels [5]. Low levels of testosterone have been associated with increased mortality in men with coronary artery disease (CAD) [6] and inverse relationship between endogenous testosterone and all-cause mortality has been reported [7].

Testosterone levels are inversely correlated with total cholesterol, LDL cholesterol, apolipoprotein B, and triglyceride and directly with HDL cholesterol. So, men with low testosterone levels have frequently metabolic syndrome [8]. Impaired endothelial function in men with hypogonadism has been reported [9]. Low levels of testosterone and free androgen index are reported in patients with CAD, atherosclerosis of the aorta or carotid atherosclerosis [10].

The testosterone level is also negatively correlated with carotid intima-media thickening [11]. Animal studies suggest that testosterone suppresses the formation of foam cells, reduces atherosclerotic lesions and has protective effects on cardiac function [12, 13].

Little is known about the possible role of serum testosterone in response to reperfusion therapy in ST-elevation myocardial infarction (STEMI) and its relationship with ST-segment recovery. In other words, the relationship between serum testosterone levels and the success rate of PPCI have not fully investigated in patients with STEMI. The present study was therefore conducted to address this issue.

## Methods

From March 2014 to March 2015, 50 men aged over 18 with acute STEMI within 12 h of the onset of the symptoms, undergoing PPCI were enrolled in this prospective study. STEMI was diagnosed based on following criteria: typical chest pain lasting more than 30 min, ST segment elevation 0.1 mV in at least two contiguous leads and elevated troponin levels. Primary PCI was done via femoral approach using a six French catheter. Exclusion criteria were: history of chronic inflammatory disease, chronic kidney disease, anti-androgenic medication use like finasteride or tamsulosin, history of prostate or testicular cancer or treatment for this condition and age under 18. All patients were given aspirin (325 mg) and clopidogrel (600 mg) on admission in the emergency room. A bolus of 100 IU/kg of IV heparin was administrated. Primary PCI including balloon angioplasty or stenting was performed according to physician’s decision. Thrombectomy device and Eptifibatide use was also dependent on the operators. Baseline coronary flow were assessed by Thrombolysis in Myocardial Infarction (TIMI) flow grading system in the infarct-related artery (IRA). Baseline clinical and demographic data including age, hypertension, diabetes mellitus, dyslipidemia, smoking habit, medication use, systolic and diastolic blood pressure at admission were recorded. Diabetes mellitus was defined as a fasting blood glucose ≥ 126 mg/dl or treatment with anti-diabetic drugs, hypertension was defined as systolic blood pressure >140 mmHg and/or diastolic blood pressure >90 mmHg or treatment with anti-hypertension drugs, and hyperlipidemia was defined as total cholesterol >200 mg/dl or low density lipoprotein >130 mg/dl. Transthoracic echocardiography was performed in all patients and left ventricular function was measured by Simpson’s rule. Venous blood samples were collected from all subjects in the emergency room before performing the PPCI and were then sent to the laboratory. Free Testosterone and Estradiol level were measured by an ELISA immunoassay (AccuBind ELISA, monobind, USA) with a measuring range between 4 and 30 pg/ml and 4–94 pg/ml respectively (Additional file 1). Other laboratory data including cardiac enzymes and lipid profile and blood count measures were also collected. The ST-segment resolution in ECG was defined by Single-lead ST segment recovery as follows: The lead with maximum ST segment elevation in admission electrocardiogram was selected and percent reduction in ST-Elevation from baseline to 60 min post-PPCI ECG was measured. In-hospital complications including death, heart failure, ventricular arrhythmia, bleeding were reported. Coronary artery disease severity was measured by SYNTAX score (Synergy between PCI with Taxus and Cardiac Surgery) [14] by two cardiologists who were unaware of the patients’ baseline clinical and laboratory data.

The patients were divided into two groups; those with a complete ST-resolution (equal or above 50%) and those with incomplete ST-resolution (below 50%). Different variables were compared between the two groups. The patients were also divided into two groups by their pre-PPCI TIMI flow, including TIMI flow 0–1 group and the TIMI flow 2–3 group, and their serum levels of free testosterone were then compared. Serum levels of free testosterone were also compared between the patients with and without hospital complications. Also, the patients were divided into two groups according to their SYNTAX score: group 1 was defined as low SYNTAX score (≤ 22) and group 2 was defined as high SYNTAX score (>22). The study was approved by local ethics committee and informed consent was obtained from all patients.

### Statistical analysis

Continuous variables were expressed as mean ± standard deviation and qualitative variables as percentages. When the variables were quantitative, after the Kolmogorov-Smirnov test was conducted, the Pearson test was used for the normally distributed variables and Spearman’s nonparametric correlation for the nonparametric variables. The independent t-test was used to compare the quantitative data and the Chi-square test to compare the qualitative data. *P*-values less than 0.05 were considered as statistically significant. Statistical analyses were performed using SPSS 17.0 software.

## Results

Of the total of 50 eligible male patients who entered the study, two were excluded, including one patient requiring emergency CABG (Coronary Artery Bypass Graft surgery) and another who died before performing the PPCI, making for a total of 48 subjects.

The mean age of the patients included was 55.54 ± 12.20 years. Complete ST-resolution was observed in 35 (72.9%) of the patients.

The basic clinical and angiographic data were compared between the patients with and without complete ST-resolutions (Table 1). The majority of the patients in both groups had a TIMI flow of 0 and 1 before PPCI and no significant differences were observed between them in terms of their clinical and angiographic characteristics. The patients with complete ST-resolutions presented more frequently within the first 6 h of symptoms, although the difference was not statistically significant.

**Table 1:** Baseline demographic, clinical and angiographic data of patients with and without complete ST-resolution

Variables		Complete ST-resolution *N* = 35 (72.9%)	Incomplete ST-resolution *N* = 13 (27.1%)	*P* value
Mean age (years ± SD)		54.9 ± 12.01	57.15 ± 13.05	0.58
Hypertension, n (%)		7 (20%)	2 (15.4%)	0.71
Diabetes Mellitus, n (%)		4 (11.4%)	2 (15.4%)	0.65
Hyperlipidemia, n (%)		4 (11.4%)	0 (0%)	0.56
Smoking, n (%)		15 (42.9%)	6 (46.2%)	0.83
Ischemic time	≤6 h	21 (60%)	4 (30.8%)	0.07
	>6 h	14 (40%)	9 (69.2%)	
Medication use on admission Aspirin, n (%)		3 (8.57%)	2 (15.38%)	0.6
Beta-blockers, n (%)		1 (2.86%)	1 (7.7%)	0.47
ACE-inhibitors, n (%)		4 (11.4%)	1 (7.7%)	0.7
Statins, n (%)		1 (2.86%)	0 (0%)	0.53
Mean Syntax score ± SD		20.48 ± 9.04	21.92 ± 9.92	0.63
Mean Systolic blood pressure ± SD, mmHg		132.20 ± 26.03	129.66 ± 24.59	0.77
Mean Diastolic blood pressure ± SD, mmHg		83.48 ± 16.22	78.16 ± 13.94	0.32
LVEF, (%) ± SD		41.61 ± 10.27	43.84 ± 4.63	0.45
Anterior MI, n (%)		19 (54.3%)	7 (53.8%)	0.97
Pre-procedural TIMI flow, n (%)				0.31
0–1		31 (88.6%)	10 (76.9%)	
2–3		4 (11.4%)	3 (23.1%)	

*ACE-inhibitor* Angiotensin converting enzyme inhibitor, *Syntax score* Synergy between PCI with Taxus and Cardiac Surgery, *LVEF* Left ventricular ejection fraction, *MI* Myocardial Infarction, *TIMI* Thrombolysis in Myocardial Infarction

According to laboratory findings presented in Table 2, the serum levels of free testosterone and peak troponin were significantly higher and high-sensitivity C-Reactive Protein (hs-CRP) level significantly lower in patients with complete ST-resolutions compared to incomplete group.

**Table 2:** The laboratory findings in patients with and without complete ST-resolution

Variable	Complete ST-resolution *N* = 35 (72.9%)	Incomplete ST-resolution *N* = 13 (27.1%)	*P* value
Free serum testosterone (pg/ml)	15.60 ± 14.23	7.29 ± 5.08	0.04
Hs-CRP (mg/dl)	3.80 ± 2.91	7.23 ± 7.06	0.02
Estradiol (pg/ml)	74.89 ± 41.01	61.45 ± 9.67	0.25
White blood cell count (10^3^/l)	10.35 ± 2.80	11.08 ± 3.85	0.46
Neutrophil count (10^3^/l)	7.36 ± 3.24	7.66 ± 1.87	0.75
Lymphocyte count (10^3^/l)	2.01 ± 1.95	2.67 ± 3.60	0.53
Hemoglobin (g/dl)	15.42 ± 1.69	14.83 ± 1.17	0.25
Platelet count (10^3^/l)	234.70 ± 83.70	195.83 ± 55.41	0.14
Blood Glucose (mg/dl)	134.62 ± 51.68	140.07 ± 54.25	0.74
Creatinine (mg/dl)	1.16 ± 0.28	1.15 ± 0.49	0.96
Peak Troponin (ng/ml)	15.57 ± 9.5	9.49 ± 8.36	0.03
Peak CK-MB (IU/l)	225.17 ± 141.62	148.15 ± 82.97	0.07
HDL cholesterol (mg/dl)	34.88 ± 9.65	33.46 ± 10.79	0.66
LDL cholesterol (mg/dl)	107.51 ± 28.31	101.75 ± 25.83	0.54
Triglycerides (mg/dl)	121.06 ± 58.77	107.07 ± 54.66	0.46

*Hs-CRP* High sensitive C-reactive protein, *CK-MB* Creatine kinase-MB isoenzyme, *HDL* High-density lipoprotein cholesterol, *LDL* Low-density lipoprotein cholesterol

In the present study, a pre-PCI TIMI flow of 0–1 was observed in 37 patients (77.1%) and TIMI flow of 2–3 in 11 cases (22.9%). The mean serum levels of free testosterone was 11.39 ± 10.79 pg/ml in the first group compared to 20.80 ± 17.77 pg/dl in the second group (*P* = 0.03). Thus, patients with a higher baseline TIMI flow had significantly higher serum levels of free testosterone.

**Fig. 1:**
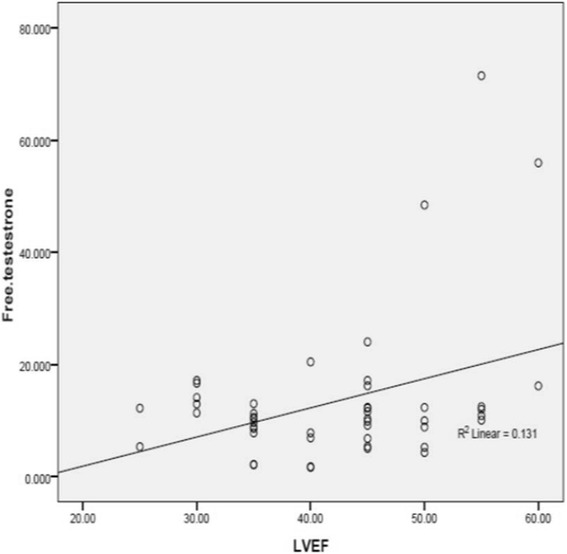
The relationship between LVEF and serum levels of free testosterone. LVEF: Left Ventricular Ejection Fraction, Patients with LVEF ≥ 40% *n* = 30 Patients with LVEF < 40% *n* = 18

LVEF was also found to be positively correlated with the serum levels of free testosterone (*r* = 0.362 and *P* = 0.01) (Fig. 1).

In-hospital complications were observed in 15 patients (31.2%), including three cases (6.3%) of ventricular arrhythmias that lead to one death, three cases of hemorrhage (6.3%) and nine cases of heart failure (18.8%).

Complications occurred in nine cases (69.2%) of incomplete ST-resolution and six cases of complete ST-resolution (17.1%); (*p* = 0.001). Thus, as expected, complication occurred more frequently in patients with failed reperfusion therapy.

**Fig. 2:**
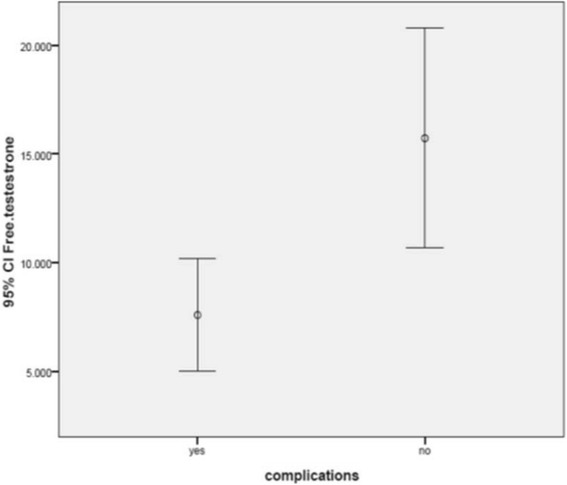
Serum free Testosterone in patients with and without complication. Patients with complications: *n* = 15, Patients without complications: *n* = 33

Moreover, lower levels of serum testosterone were observed in patients with complications than in those without complications. (7.58 ± 4.48 pg/ml vs 15.72 ± 14.49 pg/ml, *p* = 0.04) (Fig. 2).

There was no significant difference between two groups regarding coronary artery involvement based on SYNTAX score. Free testosterone levels were not significantly different between low SYNTAX score group and High SYNTAX score group (14.18 ± 12.46 vs 9.41 ± 3.54 pg/ml, *P* = 0.13 respectively).

## Discussion

The main findings of the present study were as follows: 1) higher testosterone level was associated with better response to mechanical reperfusion in patients with STEMI as manifested by complete ST-segment resolution; 2) higher testosterone level also was associated with better baseline coronary TIMI flow; 3) hypotestosteronemia was more frequent among patients with complicated STEMI; 4) better LV function was seen in patients with higher baseline testosterone levels undergoing primary PCI.

Previous studies have shown that reduced serum testosterone levels are associated with increased prothrombotic factors such as fibrinogen and factor VII [15, 16]. The potential mechanism of reperfusion failure in our patients may be related to high thrombus burden and prothrombotic state associated with low testosterone levels. Although we did not assess the thrombus burden, multiple studies have linked increased thrombus burden to reperfusion failure [17]. The other possible mechanism may be anti-inflammatory effects of testosterone [18]. Like previous studies, the present study found significantly higher levels of hs-CRP in patients with reperfusion failure compared to those with a successful reperfusion, which may be related to anti-inflammatory effects of high testosterone levels in patients with successful PPCI. Better baseline coronary blood flow in patients with higher testosterone level may be explained by direct vasodilatory effect of testosterone and improved coronary endothelial function due to more nitric oxide generation by testosterone [19, 20]. In our study, patients with complete ST-segment resolution had higher peak troponin levels. This may be explained by “washout phenomenon”: Successful reperfusion usually results in a higher and earlier release of proteins of necrosis (cardiac enzymes) into the circulation compared to patients with failed reperfusion [21–24].

Recently, Niccoli et al. examined the link between hypotestosteronemia and coronary microvascular obstruction (MVO) in patients with STEMI and revealed a higher prevalence of hypotestosteronemia in patients with STEMI compared to those with stable angina as well as in STEMI patients with MVO after PCI suggesting the potential effect of testosterone deficiency in the pathogenesis of MVO [25]. In their study, lower levels of testosterone in STEMI patients was associated with reduced success rate of PPCI as mentioned by lower post PCI TIMI flow. In contrast to their study, we assessed baseline TIMI flow not post PCI coronary flow. Previous studies have shown strong association between poor baseline coronary TIMI flow and worse outcome of patients with STEMI undergoing PPCI and less favorable response to mechanical reperfusion [26–28]. On the other hand, hypoandrogenism in men is associated with lower intrinsic fibrinolytic activity [29]. So, lower preprocedural TIMI flow and less spontaneous reperfusion in our study may be related to impaired baseline fibrinolytic properties mediated by lower testosterone level. The results obtained in Niccoli’s study on ST-resolution are similar to those obtained in the present study; however, their study didn’t assess LVEF and its relationship with testosterone levels, while the present study revealed a positive relationship between left ventricular systolic function and serum testosterone level. Nevertheless, the present study did not include patients with stable angina and didn’t measure LH and FSH level. Numerous studies have shown successful ST-resolution following reperfusion is associated with improved short-term and long-term outcomes in patients with STEMI [30]. Like previous studies, lesser in-hospital complications were observed in patients with successful ST-resolutions.

Similar to our study, Militaru et al. has recently shown negative association between 30-day mortality rate and serum levels of free testosterone in patients hospitalized with acute myocardial infarction [31]. However, their study included both STEMI and NSTEMI (Non-ST segment elevation myocardial infarction) patients that have a relatively different pathophysiologic mechanism and outcome. Also, we assessed only in-hospital and not 30-day complications.

Another study by Wickramatilake et al. found that low levels of serum testosterone are associated with in-hospital complications in patients with STEMI, which is consistent with the present study results [32]. In their study, nearly half of patients developed in-hospital complications. Unlike our study, main reperfusion strategy in their patients was thrombolysis which may account for relatively higher complication rates compared to our patients who underwent primary angioplasty.

In addition, Ohlsson et al. found a significant correlation between serum testosterone levels and the risk of cardiovascular events, as lower testosterone levels were associated with a higher risk of these events [33]. We found no association between testosterone levels and severity of coronary artery disease (CAD) as measured by SYNTAX score. Previous studies have shown conflicting results. Like our study, Davoodi et al. didn’t find any correlation between free testosterone levels and severity of CAD as assessed by Gensini score [34]. In contrary, Li et al. reported negative association between Gensini score and testosterone levels [35]. Smaller sample size and ethnical differences may explain our different results compared to this study.

Our study results could be challenged by multiple previous studies showing adverse cardiovascular effects of exogenous testosterone supplemental prescription. “TOM” trial which enrolled older men with limited mobility to testosterone gel or placebo terminated early due to higher adverse cardiovascular events in testosterone group [36]. Another retrospective cohort study on men with low testosterone showed that testosterone therapy was associated with increased risk of myocardial infarction, stroke and all-cause mortality [37]. Also, there was increased risk of non-fatal myocardial infarction in the 90 days following the initial prescription of testosterone [38]. So, in interpreting the results of present study, deleterious effect of testosterone and androgens on cardiovascular and coagulation system should be considered. It has been shown that testosterone therapy increases human platelet thromboxane A2 receptor density and aggregation responses [39] and androgen exposure is associated with increased human monocyte adhesion to endothelial cells, a proatherogenic phenomenon [40]. Also, dihydrotestosterone increases smooth muscle proliferation and expression of vascular cell adhesion molecule 1, which enhances monocyte activation in the endothelium and promotes atherosclerosis [41].

On the other hand, low testosterone level may be the consequence of acute myocardial infarction and advanced CAD. It is well known that acute stress is associated with transient hypogonadism and low testosterone level [42]. So, in present study, more severe in-hospital complications may be associated with greater stress and hence lower free testosterone level.

The present study also found that the serum level of free testosterone is directly related with LVEF, as higher levels of serum testosterone were associated with a better left ventricular function. The exact pathophysiological mechanism of this relationship is yet to be defined; however, a number of potential mechanisms may be responsible. Several animal studies have shown that testosterone may play a role in cardioprotection following ischemia/reperfusion phenomenon. Liu et al. demonstrated that testosterone administration can reduce the degree of apoptosis in animals with ischemia/reperfusion [43]. Another animal study showed that testosterone causes resistance to ischemia and reduces myocyte death following myocardial infarction (MI) through activating the mitochondrial ATP-dependent K+ channels (mitoKATP) [44]. Based on the findings of these and other animal studies [45, 46] high levels of testosterone in acute myocardial infarction (AMI) may be associated with resistance to reperfusion injury, apoptosis and cell death, thereby preventing the extensive formation of left ventricular myocardial necrosis and ultimately improving LVEF. The possible anti-inflammatory effects of testosterone on mechanisms involved in the development and progression of congestive heart failure may also improve LVEF in patients with high levels of testosterone as shown in animal models [47]. Nevertheless, no positive relationships were reported between testosterone levels and LVEF in a previous study in patients with CHF [48]. The present study may be the first to suggest that a direct relation exists between LVEF and serum testosterone levels.

The limitations of the present study include the small sample size, single-center study and the lack of long-term follow-up of the patients. Therefore, the results of present study need to be confirmed in larger multicenter studies and the results of the current study should be interpreted with caution regarding any cause-effect relationship between low testosterone levels and outcome of patients with STEMI undergoing reperfusion therapy and possible deleterious effects of testosterone on cardiovascular system should be considered.

## Conclusion

Based on the results of the present study, higher levels of serum testosterone in men with STEMI undergoing PPCI are associated with a better response to mechanical reperfusion, fewer complications and a better left ventricular function. According to the findings of the present study, it may be prudent to measure androgen profile in men with poor response to reperfusion therapy in the setting of acute STEMI.

## Additional file

Additional file 1: Free Testosterone kit datasheet by Monobind Inc. (DOCX 152 kb)

## Abbreviations

AMI: Acute myocardial infarction
CABG: Coronary Artery Bypass Graft surgery
CAD: Coronary artery disease
CHF: Congestive heart failure
CVD: Cardiovascular disease
HDL: High-density lipoprotein
hs-CRP: high-sensitivity C-reactive protein
IRA: Infarct-related artery
LDL: Low-density lipoprotein
LVEF: Left ventricular ejection fraction
MI: Myocardial infarction
MVO: Microvascular obstruction
NSTEMI: Non-ST-elevation myocardial infarction
PCI: Percutaneous coronary intervention
PPCI: Primary Percutaneous coronary intervention
STEMI: ST-segment elevation myocardial infarction
SYNTAX: Synergy between PCI with Taxus and Cardiac Surgery
TIMI: Thrombolysis in Myocardial Infarction
