# Supplementary material for: Association of admission testosterone level with ST-segment resolution in male patients with ST-segment elevation myocardial infarction undergoing primary percutaneous coronary intervention
Source: Basic Clin Androl. 2017 Jul 21;27:14. doi: 10.1186/s12610-017-0058-7 (PMC5532774; doi:10.1186/s12610-017-0058-7)
Supplement: Additional file 1: — Free Testosterone kit datasheet by Monobind Inc. (DOCX 152 kb) [file 12610_2017_58_MOESM1_ESM.docx]

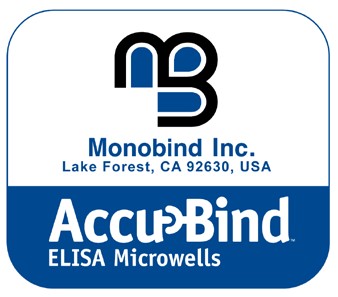


**Free Testosterone Test System**

***Product Code: 5325-300***

# 1.0 INTRODUCTION

## Intended Use: The Quantitative Determination of Free Testosterone Concentration in Human Serum or Plasma by a Microplate Enzyme Immunoassay, Colorimetric

**2.0 SUMMARY AND EXPLANATION OF THE TEST**

Testosterone, (17β-Hydroxy-4-androstene-3-one), a C19 steroid, is the most potent naturally secreted androgen.1 In normal post

pubertal males, testosterone is secreted primarily by the testes, with only a small amount derived from peripheral conversion of 4- Androstene-3, 17-dione (ASD).2 In adult women, it has been estimated that over 50% of serum testosterone is derived from

peripheral conversion of ASD secreted by the adrenal and ovary, with the remainder from direct secretion of testosterone by these glands.

In the male, testosterone is mainly synthesized in the interstitial Leydig cells and the testis, and is regulated by the interstitial cell stimulating hormone (ICSH), or luteinizing hormone (LH) of the

anterior pituitary (the female equivalent of ICSH).3 Testosterone is responsible for the development of secondary sex characteristics,

such as the accessory sex organs, the prostate, seminal vesicles and the growth of facial, pubic and auxiliary hair. Testosterone measurements have been very helpful in evaluating hypogonadal states. Increased testosterone levels in males can be found in complete androgen resistance (testicular feminization). Common causes of decreased testosterone levels in males include: hypogonadism, orchidectomy, estrogen therapy, Klinefelter's

syndrome, hypopituitarism, and hepatic cirrhosis.2-4

In the female, testosterone levels are normally found to be much lower than those encountered in the healthy male. Testosterone in the female comes from three sources. It is secreted in small quantities by both the adrenal glands and the ovaries, and in healthy women, 50–60% of the daily testosterone production arises from peripheral metabolism of prohormone, chiefly androstenedione. Common causes of increased serum testosterone levels in females include polycystic ovaries (Stein- Leventhal syndrome), ovarian tumors, adrenal tumors and adrenal hyperplasia. Virilization in women is associated with the administration of androgens and endogenous overproduction of testosterone. There appears to be a correlation between serum testosterone levels and the degree of virilization in women, although approximately 25% of women with varying degrees of virilism have serum testosterone levels that fall within the female reference range.

The majority of testosterone is bound to transport proteins: weakly bound to albumin and cortisol binding protein (25-65% females; 45-

85% males) and tightly bound to sex hormone-binding globulin (SHBG) (35-75% females; 14-50% males).8 A small fraction exist as unbound or free testosterone; however, this form is biologically active.

Therefore, the free hormone concentration is a better indicator of biological activity than total testosterone.

# 3.0 PRINCIPLE

## Competitive Enzyme Immunoassay (TYPE 7):

The essential reagents required for an enzyme immunoassay include antibody, enzyme-antigen conjugate and native antigen.

Upon mixing biotinylated antibody, enzyme-antigen conjugate and a serum containing the native free antigen, a competition reaction results between the native free antigen and the enzyme-antigen conjugate for a limited number of antibody binding sites. The interaction is illustrated by the followed equation:

ka

EnzAg + Ag + AbBtn
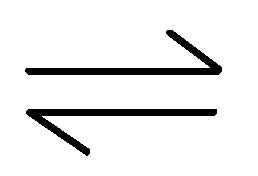
 AgAbBtn + EnzAgAbBtn

k-a

AbBtn= Biotinylated Antibody (Constant Quantity) Ag = Native Antigen (Variable Quantity)

**Enz**Ag = Enzyme-antigen Conjugate (Constant Quantity) AgAbBtn = Antigen-Antibody Complex

**Enz**Ag AbBtn = Enzyme-antigen Conjugate -Antibody Complex ka = Rate Constant of Association

k-a = Rate Constant of Disassociation K = ka / k-a = Equilibrium Constant

A simultaneous reaction between the biotin attached to the

antibody and the streptavidin immobilized on the microwell occurs. This effects the separation of the antibody bound fraction after decantation or aspiration.

AgAbBtn + EnzAgAbBtn + StreptavidinCW ⇒ immobilized complex StreptavidinCW = Streptavidin immobilized on well

Immobilized complex = sandwich complex bound to the solid surface

The enzyme activity in the antibody bound fraction is inversely proportional to the native antigen concentration. By utilizing several different serum references of known antigen concentration, a dose response curve can be generated from which the antigen concentration of an unknown can be ascertained.

# 4.0 REAGENTS

## Materials Provided:

1. **Free Testosterone Calibrators* – 1ml/vial - Icons A-G**

Seven (7) vials of serum reference for Free Testosterone at

**approximate*** concentrations of 0 (**A**), 0.2 (**B**), 1.0 (**C**)**,** 2.1 (**D**),

5.6 (**E**), 13.8 (**F**) and 37.5 (**G**) in pg/ml. Store at 2-8°C. A preservative has been added. The calibrators can be expressed in molar concentrations (pM/L) by multiplying by

3.47. For example: 1pg/ml x 3.47 = 3.47 pM/L

* Exact levels are given on the labels on a lot specific basis.

**E**

## Free Testosterone Enzyme Reagent – 6ml/vial

One (1) vial of Testosterone (Analog)-horseradish peroxides (HRP) conjugate in a protein stabilizing matrix with yellow dye. Store at 2-8°C.

## Free Testosterone Biotin Reagent – 6ml/vial - Icon ∇

One (1) vial of reagent contains anti-Testosterone biotinylated purified rabbit IgG conjugate in buffer, blue dye and preservative. Store at 2-8°C.

## Streptavidin Coated Plate – 96 wells – Icon ⇓

One 96-well microplate coated with 1.0 µg/ml streptavidin and packaged in an aluminum bag with a drying agent. Store at 2-8°C.

## Wash Solution Concentrate – 20ml/vial – Icon
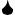


One (1) vial containing a surfactant in buffered saline. A preservative has been added. Store at 2-8°C.

## Substrate A – 7ml/vial - Icon SA

One (1) vial contains tetramethylbenzidine (TMB) in buffer. Store at 2-8°C. See “Reagent Preparation.”

## Substrate B – 7ml/vial - Icon SB

One (1) vial contains hydrogen peroxide (H2O2) in buffer. Store at 2-8°C. See “Reagent Preparation.”


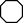


**STOP**

## Stop Solution - 8ml/vial - Icon

One (1) vial contains a strong acid (1N HCl). Store at 2-8°C.

## Product Instructions

**Note 1:** Do not use reagents beyond the kit expiration date.

**Note 2:** Avoid extended exposure to heat and light. **Opened reagents are stable for sixty (60) days when stored at 2-8°C. Kit and component stability are identified on label.**

**Note 3:** Above reagents are for a single 96-well microplate.

## 4.1 Required But Not Provided:

1. Pipette capable of delivering 0.020 & 0.050ml (20µl & 50µl) volumes with a precision of better than 1.5%.
2. Dispenser(s) for repetitive deliveries of 0.100 & 0.350ml (100 & 350µl) volumes with a precision of better than 1.5%.
3. Adjustable volume (200-1000µl) dispenser(s) for conjugate.
4. Microplate washer or a squeeze bottle (optional).
5. Microplate Reader with 450nm and 620nm wavelength absorbance capability.
6. Absorbent Paper for blotting the microplate wells.
7. Plastic wrap or microplate cover for incubation steps.
8. Vacuum aspirator (optional) for wash steps.
9. Timer.
10. Quality control materials.

# 5.0 PRECAUTIONS

## For In Vitro Diagnostic Use

**Not for Internal or External Use in Humans or Animals**

All products that contain human serum have been found to be non- reactive for Hepatitis B Surface Antigen, HIV 1&2 and HCV Antibodies by FDA required tests. Since no known test can offer complete assurance that infectious agents are absent, all human serum products should be handled as potentially hazardous and capable of transmitting disease. Good laboratory procedures for handling blood products can be found in the Center for Disease Control / National Institute of Health, "Biosafety in Microbiological and Biomedical Laboratories," 2nd Edition, 1988, HHS Publication No. (CDC) 88-8395.

## Safe Disposal of kit components must be according to local regulatory and statutory requirement.

**6.0 SPECIMEN COLLECTION AND PREPARATION**

The specimens shall be blood, serum or plasma in type, and the usual precautions in the collection of venipuncture samples should be observed. For accurate comparison to established normal values, a fasting morning serum sample should be obtained. The blood should be collected in a plain redtop venipuncture tube or (for plasma) in evacuated tube(s) containing heparin. Allow the blood to clot for serum samples. Centrifuge the specimen to separate the serum or plasma from the cells.

Samples may be refrigerated at 2-8oC for a maximum period of five

(5) days. If the specimen(s) cannot be assayed within this time, the sample(s) may be stored at temperatures of -20oC for up to 30 days. Avoid use of contaminated devices. Avoid repetitive freezing and thawing. When assayed in duplicate, 0.040ml (40µl) of the specimen is required.

# 7.0 QUALITY CONTROL

Each laboratory should assay controls at levels in the low, normal and high range for monitoring assay performance. These controls should be treated as unknowns and values determined in every test procedure performed. Quality control charts should be maintained to follow the performance of the supplied reagents. Pertinent statistical methods should be employed to ascertain trends. The individual laboratory should set acceptable assay performance limits. In addition, maximum absorbance should be consistent with past experience. Significant deviation from established performance can indicate unnoticed change in experimental conditions or degradation of kit reagents. Fresh reagents should be used to determine the reason for the variations.

# 8.0 REAGENT PREPARATION

## Wash Buffer

Dilute contents of wash solution to 1000ml with distilled or deionized water in a suitable storage container. Diluted buffer can be stored at 2-30°C for up to 60 days.

1. **Working Substrate Solution -** Stable for 1 year.

Pour the contents of the amber vial labeled Solution ‘A’ into the clear vial labeled Solution ‘B’. Place the yellow cap on the

clear vial for easy identification. Mix and label accordingly. Store at 2 - 8°C.

### Note1 : Do not use the working substrate if it looks blue.

***Note 2: Do not use reagents that are contaminated or have bacteria growth.***

**9.0 TEST PROCEDURE**

*Before proceeding with the assay, bring all reagents, serum reference calibrators and controls to room temperature (20 - 27°C).*

### **Test Procedure should be performed by a skilled individual or trained professional**

1. Format the microplates’ wells for each serum reference, control and patient specimen to be assayed in duplicate. **Replace any unused microwell strips back into the aluminum bag, seal and store at 2-8°C.**
2. Pipette 0.020ml (20µL) of the appropriate serum reference, control or specimen into the assigned well.
3. Add 0.050ml (50µl) of the Free Testosterone Enzyme Reagent to all wells.
4. Swirl the microplate gently for 20-30 seconds to mix.
5. Add 0.050 ml (50µl) of Free Testosterone Biotin Reagent to all wells.
6. Swirl the microplate gently for 20-30 seconds to mix.
7. Cover and incubate for 60 minutes at room temperature.
8. Discard the contents of the microplate by decantation or aspiration. If decanting, blot the plate dry with absorbent paper.
9. Add 0.350ml (350µl) of wash buffer (see Reagent Preparation Section), decant (tap and blot) or aspirate. Repeat two (2) additional times for a total of three (3) washes. **An automatic or manual plate washer can be used. Follow the manufacturer’s instruction for proper usage. If a squeeze bottle is employed, fill each well by depressing the container (avoiding air bubbles) to dispense the wash. Decant the wash and repeat two (2) additional times.**
10. Add 0.100ml (100µl) of working substrate solution to all wells (see Reagent Preparation Section). **Always add reagents in the same order to minimize reaction time differences between wells.**

**DO NOT SHAKE THE PLATE AFTER SUBSTRATE ADDITION**

1. Incubate at room temperature for fifteen (15) minutes.
2. Add 0.050ml (50µl) of stop solution to each well and gently mix for 15-20 seconds. **Always add reagents in the same order to minimize reaction time differences between wells.**
3. Read the absorbance in each well at 450nm (using a reference wavelength of 620-630nm to minimize well imperfections) in a microplate reader. **The results should be read within thirty**

## (30) minutes of adding the stop solution.

**10.0 CALCULATION OF RESULTS**

**A dose response curve is used to ascertain the concentration of Free Testosterone in unknown specimens.**

1. Record the absorbance obtained from the printout of the microplate reader as outlined in Example 1.
2. Plot the absorbance for each duplicate serum reference versus the corresponding Free Testosterone concentration in pg/ml on linear graph paper (do not average the duplicates of the serum references before plotting).
3. Connect the points with a best-fit curve.
4. To determine the concentration of Free Testosterone for an unknown, locate the average absorbance of the duplicates for each unknown on the vertical axis of the graph, find the intersecting point on the curve, and read the concentration (in pg/ml) from the horizontal axis of the graph (the duplicates of the unknown may be averaged as indicated). In the following example, the average absorbance 1.113 intersects the dose response curve at (1.56pg/ml) Free Testosterone concentration (See Figure 1).

**Note:** Computer data reduction software designed for ELISA assays may also be used for the data reduction. If such software is utilized, the validation of the software should be ascertained.

## EXAMPLE 1

| **Sa****mple I.D.** | **Well Number** | **Abs (A)** | **Mean Abs (B)** | **Value (pg/ml)** |
| --- | --- | --- | --- | --- |
| **Cal A** | A1 | 2.605 | 2.578 | 0 |
|  | B1 | 2.561 |  |  |
| **Cal B** | C1 | 2.387 | 2.320 | 0.20 |
|  | D1 | 2.252 |  |  |
| **Cal C** | E1 | 1.447 | 1.446 | 1.00 |
|  | F1 | 1.444 |  |  |
| **Cal D** | G1 | 0.948 | 0.934 | 2.13 |
|  | H1 | 0.920 |  |  |
| **Cal E** | A2 | 0.530 | 0.517 | 5.64 |
|  | B2 | 0.504 |  |  |
| **Cal F** | C2 | 0.328 | 0.316 | 13.80 |
|  | D2 | 0.304 |  |  |
| **Cal G** | G2 | 0.184 | 0.182 | 37.50 |
|  | H2 | 0.180 |  |  |
| **Patient** | A3 | 1.071 | 1.113 | 1.56 |
|  | B3 | 1.154 |  |  |

*The data presented in Example 1 and Figure 1 is for illustration only and **should not** be used in lieu of a standard curve prepared with each assay.

**Figure 1**

3.00

Patient

2.50

**Absorbance(s)**

2.00

1.50

1.00

0.50

0.00

0 10 20 30 40

**Free Testosterone Values in pg/ml**

# 11.0 Q.C. PARAMETERS

## In order for the assay results to be considered valid the following criteria should be met:

1. Failure to remove adhering solution adequately in the aspiration or decantation wash step(s) may result in poor replication and spurious results.
2. Use components from the same lot. No intermixing of reagents from different batches.
3. Accurate and precise pipetting, as well as following the exact time and temperature requirements prescribed are essential. Any deviation from Monobind’s IFU may yield inaccurate results.
4. All applicable national standards, regulations and laws, including, but not limited to, good laboratory procedures, must be strictly followed to ensure compliance and proper device usage.
5. It is important to calibrate all the equipment e.g. Pipettes, Readers, Washers and/or the automated instruments used with this device, and to perform routine preventative maintenance.
6. Risk Analysis- as required by CE Mark IVD Directive 98/79/EC - for this and other devices, made by Monobind, can be requested via email from [Monobind@monobind.com.](mailto:Monobind@monobind.com)

## 12.2 Interpretation

### Measurements and interpretation of results must be performed by a skilled individual or trained professional.

1. Laboratory results alone are only one aspect for determining patient care and should not be the sole basis for therapy, particularly if the results conflict with other determinants.
2. The reagents for the test system procedure have been formulated to eliminate maximal interference; however, potential interaction between rare serum specimens and test reagents can cause erroneous results. Heterophilic antibodies often cause these interactions and have been known to be problems for all kinds of immunoassays. (*Boscato LM Stuart MC. ‘Heterophilic antibodies: a problem for all immunoassays’ Clin.Chem. 1988:3427-33).* For diagnostic purposes, the results from this assay should be used in combination with clinical examination, patient history, and all other clinical findings.
3. For valid test results, adequate controls and other parameters must be within the listed ranges and assay requirements.
4. If test kits are altered, such as by mixing parts of different kits, which could produce false test results, or if results are incorrectly interpreted, Monobind shall have no liability.
5. If computer controlled data reduction is used to interpret the results of the test, it is imperative that the predicted values for the calibrators fall within 10% of the assigned concentrations.

# 13.0 EXPECTED RANGES OF VALUES

In agreement with established reference intervals5 for a “normal“ adult population, the expected ranges for the Free Testosterone AccuBind® ELISA Test System are detailed in Table 1.

## TABLE I

**Expected Values for the Free Testosterone Test System (pg/ml)**

**TABLE 2**

Within Assay Precision (Values in pg/ml )

| **Size** | | **96(A)** | **192(B)** |
| --- | --- | --- | --- |
| **Reagent (fill)** | **A)** | **1ml set** | **1ml set** |
|  | **B)** | **1 (6ml)** | **2 (6ml)** |
|  | **C** | **1 (6ml)** | **2 (6ml)** |
|  | **D** | **1 plate** | **2 plates** |
|  | **E** | **1 (20ml)** | **1 (20ml)** |
|  | **F)** | **1 (7ml)** | **2 (7ml)** |
|  | **G)** | **1 (7ml)** | **2 (7ml)** |
|  | **H)** | **1 (8ml)** | **2 (8ml)** |

| **Sample** | **N** | **X** | **σ** | **C.V.%** |
| --- | --- | --- | --- | --- |
| **Low** | 24 | 2.87 | 0.21 | 7.4 |
| **Normal** | 24 | 23.10 | 1.55 | 6.7 |
| **High** | 24 | 38.59 | 1.08 | 2.8 |

## TABLE 3

Between Assay Precision (Values in pg/ml )

| **Sample** | **N** | **X** | **σ** | **C.V.%** |
| --- | --- | --- | --- | --- |
| **Low** | 24 | 3.29 | 0.33 | 10.1 |
| **Normal** | 24 | 23.95 | 2.39 | 9.9 |
| **High** | 24 | 36.79 | 2.06 | 5.6 |

*As measured in ten experiments in duplicate over a ten day period.

## Sensitivity


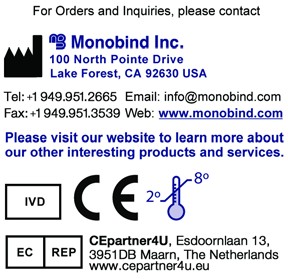
The Free Testosterone AccuBind® ELISA Test System has a sensitivity of 0.0019 pg/well. This is equivalent to a sample containing a concentration of 0.095pg/ml. The sensitivity was ascertained by determining the variability of the 0 ng/ml serum calibrator and using the 2σ (95% certainty) statistic to calculate the minimum dose.

## Specificity

The % cross reactivity of the Free Testosterone antibody to selected substances was evaluated by adding the interfering substance to a serum matrix at various concentrations. The cross- reactivity was calculated by deriving a ratio between dose of interfering substance to dose of Testosterone needed to displace the same amount of labeled analog.

| Substance | Cross Reactivity |
| --- | --- |
| Testosterone | 1.0000 |
| Androstenedione | 0.0009 |
| Dihydotestosterone | 0.0178 |
| Cortisone | <0.0001 |
| Corticosterone | <0.0001 |
| Cortisol | <0.0001 |
| Spirolactone | <0.0001 |
| Progesterone | <0.0001 |
| 17α-OH Progesterone | <0.0001 |
| DHEA sulfate | <0.0001 |
| Estradiol | <0.0001 |
| Estrone | <0.0001 |
| Estriol | <0.0001 |
| Hemolysis | <0.0001 |
| Rubella | <0.0001 |
| Lipemia | <0.0001 |

# 15.0 REFERENCES

1. Dorfman, RI and Shipley, RA, ED: Androgens, New York,: John Wiley and Sons, 1956.
2. Horton R, Tait JF: Androstenedione production and
3. The absorbance (OD) of calibrator 0 pg/ml should be > 1.3.

| **Male** | 4.0 – 30.0 |
| --- | --- |
| **Female** | 0.4 – 7.1 |

1. Four out of six quality control pools should be within the

interconversion rates measured in peripheral blood and studies on the possible site of conversion to testosterone. J.Clin Invest

established ranges.

# RISK ANALYSIS

*The MSDS and Risk Analysis Form for this product is available on request from Monobind Inc.*

## Assay Performance

1. It is important that the time of reaction in each well is held constant to achieve reproducible results.
2. Pipetting of samples should not extend beyond ten (10) minutes to avoid assay drift.
3. Highly lipemic, hemolyzed or grossly contaminated specimen(s) shou ld not be used.
4. If more than one (1) plate is used, it is recommended to repeat the dose response curve.
5. The addition of substrate solution initiates a kinetic reaction, which is terminated by the addition of the stop solution. Therefore, the substrate and stop solution should be added in the same sequence to eliminate any time-deviation during reaction.
6. Plate readers measure vertically. Do not touch the bottom of the wells.

It is important to keep in mind that establishment of a range of

values, which can be expected to be found by a given method for a population of "normal” persons, is dependent upon a multiplicity of factors: the specificity of the method, the population tested and the precision of the method in the hands of the analyst. For these reasons, each laboratory should depend upon the range of expected values established by the Manufacturer only until an in- house range can be determined by the analysts using the method with a population indigenous to the area in which the laboratory is located.

# PERFORMANCE CHARACTERISTICS

## Precision

The within and between assay precision of the Free Testosterone AccuBind® ELISA Test System were determined by analyses on three different levels of pool control sera. The number, mean values, standard deviation and coefficient of variation for each of these control sera are presented in Table 2 and Table 3.

45: 301-303, 1966.

1. Faiman C and Winter, JSD, Reyes, FI, Clin *Obstet Gynaecol,* 3, 467 (1976).
2. Sizonenka, PC, *Pediatrician,* 14, 191 (1987).
3. Cummings DC, Wall SR: Non sex hormone binding globulin bound testosterone as a marker for hyperandrogenism. J. Clin Endocrinol Metab. 61:873-876, 1985.
4. Lashansky, G, et. al., *J Clin Endocrinol Metab*,58, 674 (1991)
5. Tietz, NW, ED: Clinical Guide to Laboratory Tests, 3rd ed. Philadelphia, WA Saunders Co, 1995.
6. Smith SW: Free Testosterone, *AACC Endo*; 11(3), 59-62 (1993).

## Revision: 4 Date: 2013-AUG-05 DCO: 0895

**Product Code: 5325*-300***
